# Risk Assessment and Personalized Treatment Options in Inherited Dilated Cardiomyopathies: A Narrative Review

**DOI:** 10.3390/biomedicines12081643

**Published:** 2024-07-24

**Authors:** Diana-Aurora Arnautu, Dragos Cozma, Ioan-Radu Lala, Sergiu-Florin Arnautu, Mirela-Cleopatra Tomescu, Minodora Andor

**Affiliations:** 1 Multidisciplinary Heart Research Center, Victor Babes University of Medicine and Pharmacy, 300041 Timisoara, Romania; aurora.bordejevic@umft.ro (D.-A.A.); tomescu.mirela@umft.ro (M.-C.T.);; 2 Department of Internal Medicine I, Victor Babes University of Medicine and Pharmacy, 300041 Timisoara, Romania; 3 Department of Cardiology, Victor Babes University of Medicine and Pharmacy, 300041 Timisoara, Romania; 4 Department of Cardiology, Western University Vasile Goldis, 310025 Arad, Romania

**Keywords:** inherited dilated cardiomyopathy, risk assessment, genotype-specific therapy

## Abstract

Considering the worldwide impact of heart failure, it is crucial to develop approaches that can help us comprehend its root cause and make accurate predictions about its outcome. This is essential for lowering the suffering and death rates connected with this widespread illness. Cardiomyopathies frequently result from genetic factors, and the study of heart failure genetics is advancing quickly. Dilated cardiomyopathy (DCM) is the most prevalent kind of cardiomyopathy, encompassing both genetic and nongenetic abnormalities. It is distinguished by the enlargement of the left ventricle or both ventricles, accompanied by reduced contractility. The discovery of the molecular origins and subsequent awareness of the molecular mechanism is broadening our knowledge of DCM development. Additionally, it emphasizes the complicated nature of DCM and the necessity to formulate several different strategies to address the diverse underlying factors contributing to this disease. Genetic variants that can be transmitted from one generation to another can be a significant contributor to causing family or sporadic hereditary DCM. Genetic variants also play a significant role in determining susceptibility for acquired triggers for DCM. The genetic causes of DCM can have a large range of phenotypic expressions. It is crucial to select patients who are most probable to gain advantages from genetic testing. The purpose of this research is to emphasize the significance of identifying genetic DCM, the relationships between genotype and phenotype, risk assessment, and personalized therapy for both those affected and their relatives. This approach is expected to gain importance once treatment is guided by genotype-specific advice and disease-modifying medications.

## 1. Introduction

Dilated cardiomyopathy (DCM) affects more than 0.4% of the general population and is the second most frequent cause of heart failure, accounting for around 36% of all heart failure patients, second only to coronary artery disease. The 5-year survival rate following a heart failure diagnosis is just 50%, and mortality might result from arrhythmias or the gradual worsening of heart failure [1]. While there have been some decreases in the occurrence of heart failure due to a decrease in risk factors for cardiovascular disease and more effective therapy, the economic cost associated with HF remains substantial [2]. Approximately 25–30% of individuals diagnosed with DCM are believed to have a hereditary factor that can be identified [3]. The exact prevalence of genetically caused DCM is unknown. It is estimated to vary from 4‰ to 0.4‰. Around 25% of individuals with idiopathic DCM have a family history of the disease. These data are obtained predominantly from European people [3].

Genetic factors that contribute to DCM are not simply caused by individual DNA variants with significant effects, as in a Mendelian model. It is likely that various mutations with unimportant individual outcomes may together contribute to the inherited cardiomyopathy. Even in acquired forms of DCM (such as those induced by alcohol abuse or chemotherapy) one cannot dismiss the possibility of a significant genetic vulnerability to the respective condition [4,5].

Hereditary DCM is classified into syndromic and non-syndromic.

The term “syndromic” is used to describe an illness that is identified by a combination of visible characteristics that either strongly indicate the diagnosis and may be verified by genetic testing or allow for diagnosis even without definitive genetic evidence [6]. 

Patients with DCM that is neither acquired (secondary) nor syndromic present a non-syndromic hereditary condition [6,7]. 

The diagnosis of non-syndromic DCM is established when at least two relatives meet the criteria for DCM, or when the patient fulfils the criteria for DCM and has a first-degree family member that died suddenly at an age < 35 years [8]. Non-syndromic DCM is typically anticipated when individuals in the proband’s family have a history of heart failure or of premature and unexpected cardiac death without a clearly identifiable reason. If an enlargement of the LV is observed without a significant decrease in LVEF at the evaluation of close relatives, it is advisable to consider a diagnosis of non-syndromic DCM [4,5]. 

Inherited DCM is frequently met as a sporadic illness in clinical practice, meaning that there is no documented familial history of DCM. The hereditary aspect of DCM, how-ever, is frequently not immediately evident. If a family history is not thoroughly obtained, DCM may be incorrectly labelled as sporadic [9]. Innovative studies conducted by the team at the Mayo Clinic throughout the 1980s and 1990s showed that by systematically screening the family members of individuals with DCM, it was possible to identify familial DCM in around 20–30% of the patients [10,11].

In a recent study, Huggins et al. examined a large dataset from many centers. They found that around 12% of the probands had familial dilated cardiomyopathy (DCM) when just the probands were included. However, when all family members were tested, the estimated frequency of familial DCM was over 30% [12]. The study was a cross-sectional multicenter study that lacked a pre-planned standardized approach and just some of the family members were evaluated. Thus, it is possible to hypothesize that the occurrence of familial DCM may have been greater if all family members at risk of the probands were carefully assessed. Similarly, conducting regular assessments throughout the years would likely result in the detection of a greater proportion of familial DCM cases due to the influence of age on the likelihood of developing the condition. The study con-ducted by Huggins et al. found that the occurrence of familial DCM was somewhat more common in Black probands compared to White probands. This might indicate the impact of mutations that enhance the manifestation of a characteristic. Additionally, it might be indicative of the demographic features of the research population and the statistical methods employed to calculate the frequency of familial DCM. The discovery should be regarded as speculative until it is tested for duplication in separate groups.

In this review, we focus on adult patients with non-syndromic inherited DCM. Our objective is to assist clinicians in comprehending the clinical significance of the hereditary DCM, identifying individuals and their relatives that could benefit from genetic evaluations, acquainting them with frequently conducted genetic tests, guiding them in understanding genetic findings, and informing them about the medical importance of these findings. 

## 2. Defining Hereditary Dilated Cardiomyopathy (DCM)

A cardiomyopathy is defined as a disease of the myocardium that is structurally and functionally abnormal, in the absence of ischemic heart disease, valvular heart disease, hypertension or congenital heart disease [4]. 

DCM is defined as the presence of LV dilatation and global or regional systolic dysfunction unexplained solely by abnormal loading conditions or coronary artery disease. In early stages, DCM may present with isolated left ventricular dilatation and normal ejection fraction [4]. Right ventricular dilatation and dysfunction may be present but are not necessary for the diagnosis. When dilatation or wall motion abnormalities are predominant to the right ventricle, the possibility of arrhythmogenic right ventricular cardiomyopathy (ARVC) should be considered [4].

Corresponding to the European Society of Cardiology Guidelines for the manage-ment of cardiomyopathies [5], left ventricular (LV) dilatation is defined by echocardiog-raphy measurements as LV end-diastolic dimensions or volumes that exceed two standard deviations over the average values for the general population, taking into account body size, sex, and/or age. In adults, this refers to an LV end-diastolic diameter of more than 58 mm in men and greater than 52 mm in females, as well as an LV end-diastolic volume index equal to or greater than 75 mL/m^2^ in males and equal to or greater than 62 mL/m^2^ in females. LV global systolic dysfunction is characterized by a left ventricular ejection fraction (LVEF) that is less than 50%. 

LV dilatation and systolic dysfunction are usually assessed from a two-dimensional (2D) echocardiogram or from cardiac magnetic resonance imaging MRI [5]. Another non-invasive methodology is myocardial nuclear research. LV ejection fractions (EFs) can also be measured using an LV angiogram [6].

Once ischemia and nonischemic causes (valvular disease, hypertension, congenital heart disease, tachyarrhythmias, thyroid disease, inflammatory or infectious diseases, radiation, toxins—most common alcohol, chemotherapeutic agents or drugs with idiosyncratic effects) have been eliminated using non-invasive assessment, most often cardiac (MRI), DCM is classified as idiopathic. In rare cases, endomyocardial biopsy is needed [5]. 

Due to the expanding accessibility of genetic testing, about 20–35% of individuals with presumed idiopathic DCM have been discovered to possess a related genetic mutation [5,6,7]. 

## 3. Diagnosis of Hereditary DCM

### 3.1. Clinical Picture

The majority of patients diagnosed with DCM are adults, with onset usually occurring in the third or fourth decade of life [9,10,11,12]. The first symptoms might be con-fusing and often do not provide enough information to distinguish it from other causes of DCM. The manifestations of illness vary from an absence of symptoms, found only during screening, to severe heart failure or life-threatening arrhythmia and unexpected cardiac death. 

Heart failure symptoms involve those of systemic and/or pulmonary congestion (dyspnea, edema), as well as of reduced cardiac output (fatigue) [6]. 

Palpitations also frequently occur as symptoms due to arrhythmias and/or conduction system disease [6]. 

Stroke or systemic embolism may develop consequent to left ventricular mural thrombus [6]. 

Peripartum cardiomyopathy, occurring during or soon after pregnancy, was once considered a rare non-genetic condition, but is now recognized as a form of familial DCM [6]. Current research indicates that up to 15% of individuals with peripartum cardiomyopathy carry pathogenic genetic mutations. For women who have genetic predisposition, pregnancy is believed to act as a trigger by causing higher stress on the circulatory system. This can lead to the manifestation of the disease at an earlier stage than it would typically occur [13,14]. Women with a positive genotype may have a lower likelihood of experiencing a reversal of DCM after pregnancy compared to those who have a negative genotype. 

Pointers that suggest a genetic cause include the occurrence of cardiomyopathy or arrhythmia before the age of 35 years, as well as a family history of DCM or sudden cardiac death. Other suggestive conditions are unexplained syncope, the association of cardiomyopathy with arrhythmia, and/or muscle disorders [15]. Ventricular and atrial arrhythmias, as well as conduction disorders, may develop when the left ventricular ejection fraction decreases [16]. 

Some individuals with inherited DCM have early or obvious abnormalities in their conduction system. When a younger individual experiences conduction disorders, it should raise the possibility of hereditary cardiomyopathy. Electrocardiogram findings may include bradycardia, atrioventricular blocks, axis deviation, or bundle branch blocks [15].

**Figure 1:**
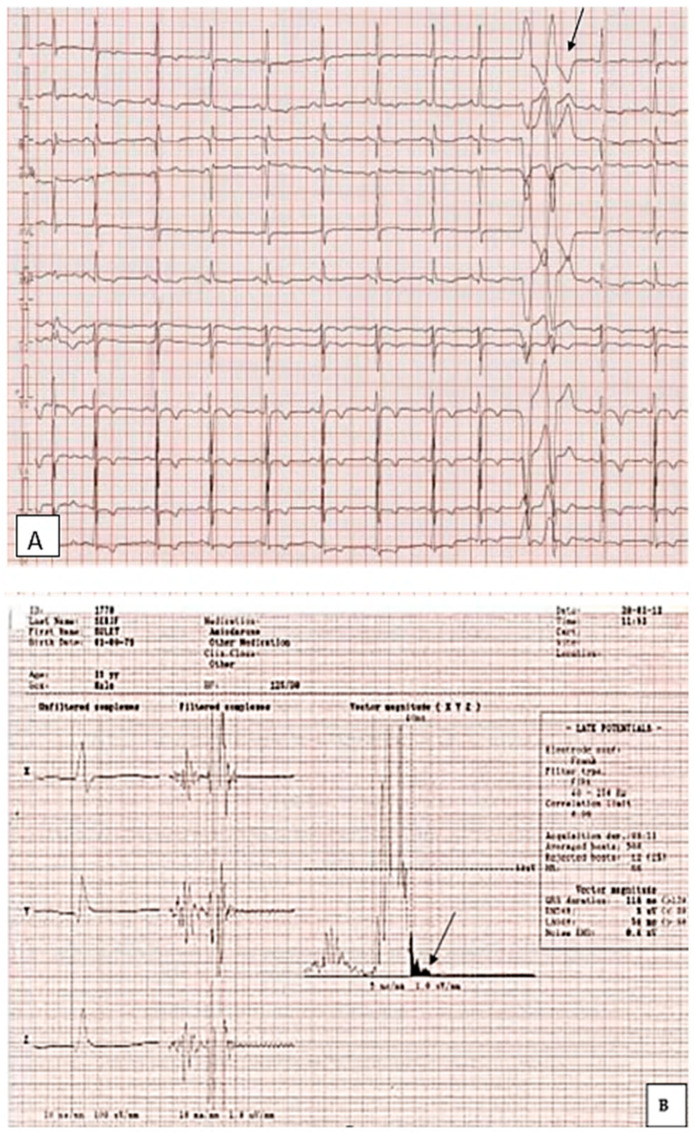
Twelve-lead electrocardiogram in a patient with familial dilated cardiomyopathy. (**A**) Sinus rhythm with recurring premature ventricular beats (arrow). Negative T waves from V1 to V6. (**B**) Ventricular late potentials (arrow).

**Figure 2:**
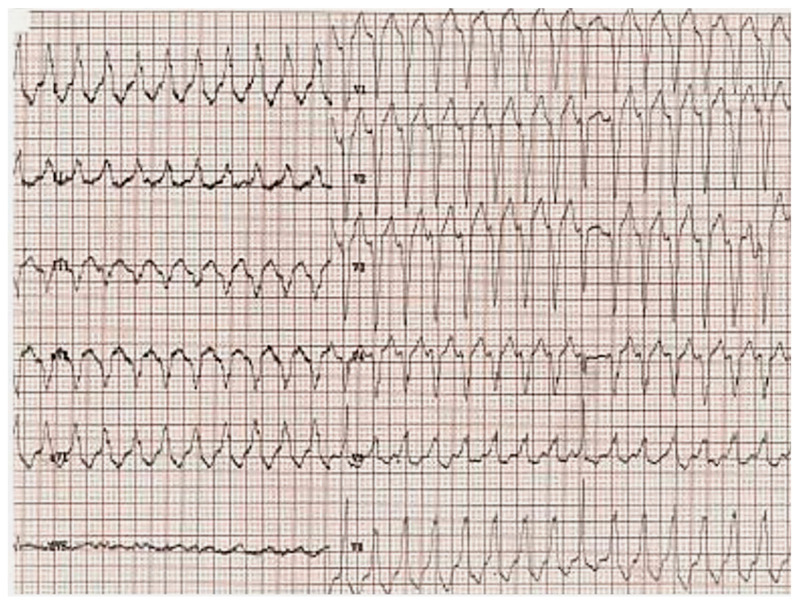
Twelve-lead electrocardiogram. Sustained ventricular tachycardia.

Figure 1 presents the ECG of an adult patient with familial dilated cardiomyopathy at sinus rhythm (A) with cardiac ventricular late potentials (B), while Figure 2 presents an episode of sustained ventricular tachycardia. 

Ventricular late potentials (VLPs) are high-frequency, low-amplitude abnormal electrical signals occurring in the terminal QRS complex or on the ST segment of the electro-cardiogram during sinus rhythm. They represent delayed conduction through a diseased myocardium and consist of the presence of electrical activity after the end of the standard QRS. The VLPs are potential substrates for reentry ventricular tachycardia.

The occurrence and intensity of cardiac arrhythmias may not be directly related to the amount of LVEF decrease. The significant similarity in genetics and physical features between arrhythmogenic DCM and arrhythmogenic right ventricular cardiomyopathy (ARVC) with left ventricular involvement has caused a lot of difficulty in diagnosis. As a result, a novel category called “arrhythmogenic cardiomyopathy” has emerged [13].

Unusual but significant warning signs such as atypical skin coloration, muscle weakness, and sensory abnormalities (such as hearing or vision loss) might suggest a particular sort of multisystem illness or a distinct genetic profile to DCM. The presence of these symptoms is sometimes referred to as “red flags” for DCM diagnosis [17]. A significant number of individuals diagnosed with inherited skeletal myopathies, such as Duchenne or Becker, experience the development of DCM as they age [18,19]. DCM can also emerge as part the clinical picture in systemic illnesses such storage or mitochondrial abnormalities [7]. 

While certain genetic variants may exhibit foreseeable trends, there can be significant variability in the manifestation of diseases, the age at which they appear, and their severity among individuals who possess variants in the same gene [20,21]. This variability can occur both within families and between different families. The penetrance of DCM can be affected by environmental variables, gene modulators, associated medical conditions, and lifestyle choices [20]. These are currently being studied. Genetic diversity may explain the variable individual vulnerability to acquired types of DCM, such as those induced by alcohol abuse or anthracycline cardiotoxicity. This complicates the distinction between hereditary and acquired myocardial diseases [12,21]. 

### 3.2. Non-Invasive Imaging Methods

They are essential for diagnosing and monitoring patients with cardiomyopathies. These methods include ultrasound-based techniques, cardiac magnetic resonance (CMR) imaging, computed tomography (CT), and nuclear techniques including positron emission tomography (PET) and scintigraphy [5].

Regular screening, including clinical evaluation, electrocardiogram (ECG), and transthoracic echocardiography (TTE), should begin in adulthood or earlier if there are treatment implications [22]. The frequency of screening can range from every six months to every five years, depending on the existence or not of high-risk indicators and the usual age at which the illness typically appears in the family [20,22]. LV dilatation on TTE in asymptomatic relatives might indicate subclinical illness, incidental illness, or physiological modifications. It is difficult to differentiate between these conditions, and since there is no dependable method to identify those who may develop obvious illness, regular surveillance is required [23]. Currently, the screening criterion in TTE continues to be the LV size and ejection fraction. However, continuing research is being conducted to explore alternative preclinical indicators. Recently, researchers have assessed the global longitudinal strain of the LV and observed a decrease in patients who are genotype-positive but phenotype-negative compared to genotype-negative controls. Yet, the exact significance of global longitudinal strain in detecting or monitoring subclinical GDCM has not been determined [5,24].

**Figure 3:**
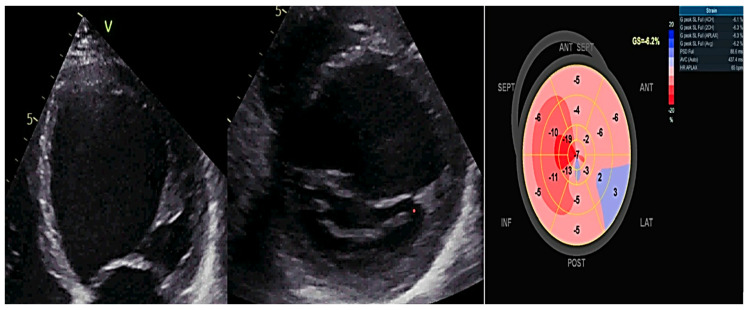
Familial dilated cardiomyopathy. Two-dimensional trans-thoracic conventional echography in apical longitudinal and parasternal short axis vi ews. Bull’s eye myocardial deformation with diffuse reduced left ventricular longitudinal peak strain patterns.

Myocardial deformation imaging, specifically using speckle tracking or tissue Doppler, is a more sensitive indicator than EF (ejection fraction) for detecting subtle ventricular dysfunction. This is particularly useful in cases such as genotype-positive HCM (hypertrophic cardiomyopathy), DCM, and ARVC (arrhythmogenic right ventricular cardiomyopathy) in family members. It also helps identify specific causes of ventricular hypertrophy, such as amyloidosis, athlete’s heart, or HCM. Mechanical dispersion serves as a sign of abnormal contraction and draws attention to subtle structural abnormalities [25,26,27,28,29]. Figure 3 exemplifies a patient with familial DCM examined by two-dimensional (2D) transthoracic conventional echocardiography and by 2D speckle-tracking echography. It shows dilated heart chambers, severe LV dysfunction (LVEF of 30%), and a Bull’s eye myocardial deformation pattern with overall low peak ventricular longitudinal strain values. 

Three-dimensional echocardiography measures, with high accuracy, the volumes of heart chambers, but it needs an adequate acoustic window. Contrast agents may be used to enhance the visibility of endocardium in order to show the existence of excessive muscle trabeculation, a condition known as hyper-trabeculation. Additionally, contrast agents can help rule out the presence of intracardiac thrombus [5]. 

CMR imaging has become the most reliable method for evaluating the size and function of the ventricles [5,24]. It should be performed in all patients recently diagnosed with cardiomyopathy. CMR can offer comprehensive anatomical and physiological data that surpass that of TTE. Additionally, CMR can provide insights into cardiac tissue features, including inflammation and fibrosis, through the use of late gadolinium enhancement (LGE). 

**Figure 4:**
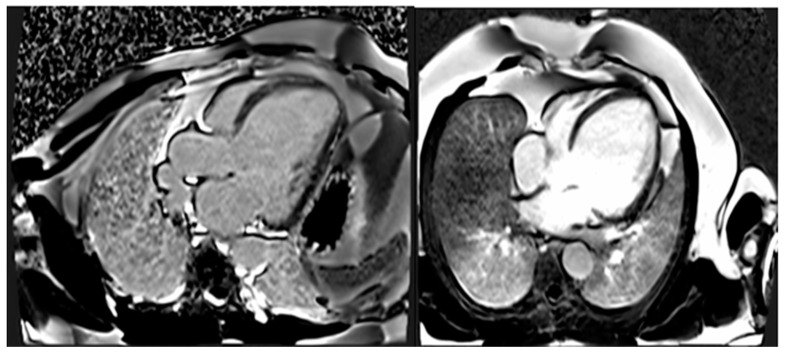
Cardiac magnetic resonance images, 4-chamber (horizontal long axis) and 2-chamber (vertical long axis). Dilated left ventricle with hyper-trabeculations of the antero-lateral wall; late gadolinium enhancement shows septal mid-wall fibrosis, suggestive of idiopathic dilative cardiomyopathy with left ventricular non-compaction.

CMR has the benefits of being non-invasive and not requiring an acoustic window while also allowing for the analysis of tissue characteristics. The latter benefit is especially crucial in diagnosing non-compaction LV cardiomyopathy (Figure 4), ARVC, myocarditis, amyloidosis, sarcoidosis, and other types of inflammatory diseases, as well as haemochromatosis [29,30]. Standard initial examination should regularly include cine imaging sequences, T2-weighted sequences, pre- and post-contrast T1 mapping, and LGE. These findings should be evaluated alongside genetic results and other clinical features by experienced professionals in cardiac imaging and heart muscle disease assessment [31]. 

Regular follow-up CMR should be conducted every 2–5 years, depending on the initial severity and clinical course of the disease. This can help assess the progression of the disease and the effectiveness of therapy [5]. The existence of LGE is linked to the possibility for arrhythmias and more severe illness [23]. Diseases connected to DSP and FLNC frequently exhibit a fibrosis pattern resembling a ring around the outer layer of the heart, known as subepicardial fibrosis [30]. This observation may suggest the need for considering the installation of an implantable cardioverter defibrillator (ICD) [31]. CMR has the capability to identify illness in persons who do not show any symptoms and may also be used to track the progression of the disease [29]. 

**Figure 5:**
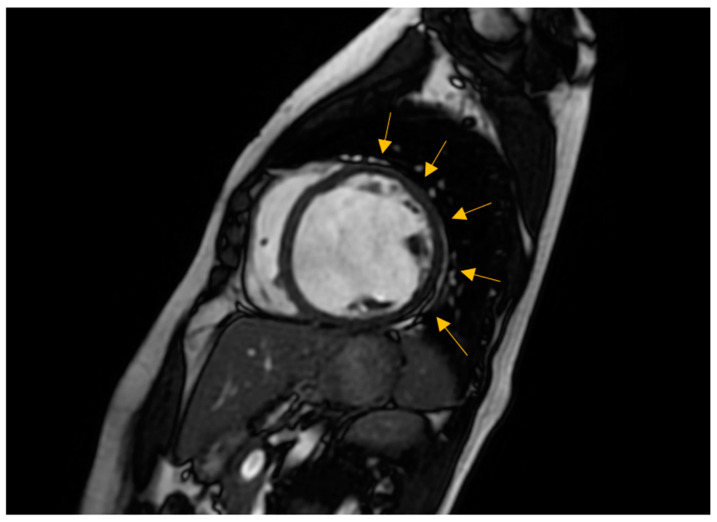
Cardiac magnetic resonance image, short axis. Dilated left ventricle with thin walls; late gadolinium enhancement highlights circumferential “ring-shape” mid wall fibrosis suggestive of desmoplakin cardiomyopathy (arrows).

The existence of LGE is linked to the possibility for arrhythmias and more severe ill-ness. Diseases connected to DSP and FLNC frequently exhibit a fibrosis pattern resembling a ring around the outer layer of the heart, known as subepicardial fibrosis, as presented in Figure 5. This observation may suggest the need for considering the installation of an implantable cardioverter defibrillator (ICD). CMR has the capability to identify illness in persons who do not show any symptoms and may also be used to track the progression of the disease [29]. 

Computed tomography-based imaging is mostly employed in patients suspected of having cardiomyopathy to exclude coronary artery disease (CAD), either as an alternative diagnosis or as a comorbidity that impacts clinical symptoms and progression [32].

Additional functional testing and imaging may be necessary on a case-by-case basis during the evaluation process.

### 3.3. Endomyocardial Biopsy

This invasive method is a topic of debate and is not currently included in the usual diagnostic process for most individuals with DCM [20].

### 3.4. Laboratory Assessment

Currently, there are no established disease-specific biomarkers for DCM [5]. However, researchers are actively investigating this area. Despite the lack of acute myocardial ischemia, high-sensitivity blood troponins frequently show aberrant levels in both acute and chronic heart failure [33]. While troponin testing may offer some risk assessment in cases of cardiomyopathy, it has a small role in the evaluation of suspected DCM [34].

Consistently high levels of serum creatinine kinase (CK) can indicate the presence of myopathies or neuromuscular illnesses such as dystrophinopathies (such as X-linked DCM or Becker muscular dystrophy), laminopathies, desminopathies, or, to a lesser extent, myofibrillar myopathy [29]. Patients with arrhythmogenic right ventricular cardiomyopathy and non-dilated LV cardiomyopathy may exhibit elevated levels of C-reactive protein, especially during bouts resembling myocarditis [35]. Increased concentrations of iron and ferritin in the blood, together with high transferrin saturation, may indicate a potential diagnosis of haemochromatosis. This should prompt additional investigation into the underlying cause (primary or secondary) by genetic testing. Leucocytopenia, lactic acidosis, and myoglobinuria may point towards the presence of mitochondrial disorders [5].

## 4. History Taking of the Initial Patient with Recently Diagnosed DCM and Family Screening

A comprehensive history taking is necessary for all patients recently diagnosed with DCM. This evaluation is crucial to ensure that any potentially curable or reversible causes are not overlooked. Nevertheless, the presence of a visible immediate factor such as alcohol abuse or chemotherapy does not rule out the possibility of a hereditary element in cardiomyopathy [5]. Women who have a genetic predisposition to peripartum cardiomyopathy may experience a faster beginning of the disease and show symptoms at a younger age compared to other members of their family [14,36]. The family’s medical history at the time of diagnosis may show no signs of illness. Regularly reviewing the family history is crucial, since it may evolve to become positive over time [7]. reference

Hereditary DCM is recognized clinically when it develops in two or more closely related individuals, after ruling out other potential causes. An exhaustive family history should encompass a three-generation genealogy [24] that investigates any instances of heart failure, arrhythmia, unexplained mortality, or other cardiac symptoms, especially if they arose at an age younger than expected. An awareness of other symptoms, such as muscular disorders, is also necessary. 

The symptoms of the family member do not necessarily have to match those of the reference patient. There are several difficulties in establishing a satisfactory heritage. The variety of illness onset and penetrance of the genetic anomaly, as well as the long period of time it takes for the disease to develop, might complicate the screening process for those who are willing to undergo it. Certain family members manifest hesitation or incapacity to undergo evaluation, while in other cases, families are limited in size and possess insufficient data. Although there are difficulties, it is crucial to create a pedigree as it aids in determining the likelihood of a genetic test being positive [24]. 

It is worth mentioning that around 25% of individuals with sporadic DCM are later identified as having familial DCM after a thorough evaluation of both the affected individual and their family members [25]. 

According to research, there is a 33% chance that a family member develops DCM or a partial phenotype of DCM by the age of 80. A partial phenotype is characterized as having either left ventricular enlargement or solely left ventricular systolic dysfunction [37]. For individuals newly diagnosed with DCM, it is recommended that their first-degree relatives have a medical assessment. This evaluation should include clinical cardiovascular screening and genetic testing. These recommendations are supported by guidelines [16,37,38,39,40]. Cardiovascular screening, which encompasses cardiovascular history, clinical examination, electrocardiogram, and cardiac imaging, serves to detect the early signs of cardiomyopathy, including asymptomatic or partial DCM phenotype cases [12]. This allows for individualized monitoring or medical treatment prior to the appearance of symptoms, with the objective of preventing the progression of DCM or premature sudden death.

It is advisable to carry out clinical screening for all unaffected first-degree relatives of any person with a genetic anomaly linked to proven or suspected familial DCM [16,41]. The screening of family members not only helps diagnose the primary case, but also often leads to the detection of the disease in persons showing no symptoms who might benefit from clinical examination. Genealogy assessment also aids in the identification of family members exhibiting pertinent phenotypic characteristics, such as isolated arrhythmias or conduction anomalies, and it allows for earlier treatment and perhaps better outcomes [42]. Additionally, it can aid in the process of family planning [43]. 

**Figure 6:**
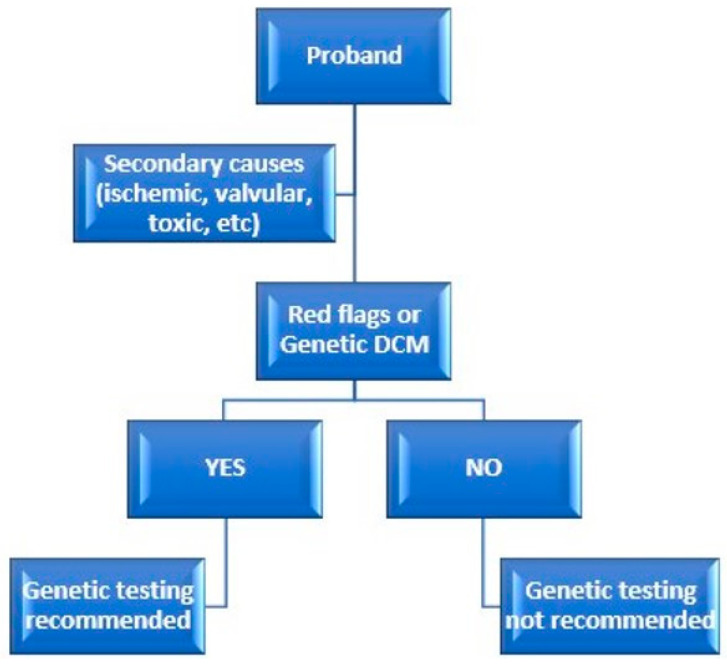
Flow-chart for assessing the risk of genetic dilated cardiomyopathy in family members. Red flags include a strong family history with 2 first-degree relatives having idiopathic dilated cardiomyopathy or sudden cardiac death at age < 35 years.

A practical flowchart for assessing the risk of genetic DCM in family members of the proband is presented in Figure 6.

## 5. Genetic Testing

Ordering genetic testing has become fairly simple, but interpreting the results can be complicated. It is recommended to undergo such testing in a specialized clinic with multidisciplinary input given the challenges in interpreting genetic variants in the absence of a con-firmed phenotypic diagnosis [23,43]. Recently, clinicians have developed techniques to assist in determining whether genetic testing should be pursued in patients with suspected genetic dilated cardiomyopathy. The Madrid Genotype Score assesses the likelihood of obtaining a meaningful genetic test outcome based on five factors: existing family history of DCM, low voltage on ECG, evidence of skeletal myopathy, lack of hypertension, and lack of left bundle branch block. When four or more of these criteria were fulfilled, a pathogenic or potentially pathogenic variation was detected in 79% of the patients [44].

**Figure 7:**
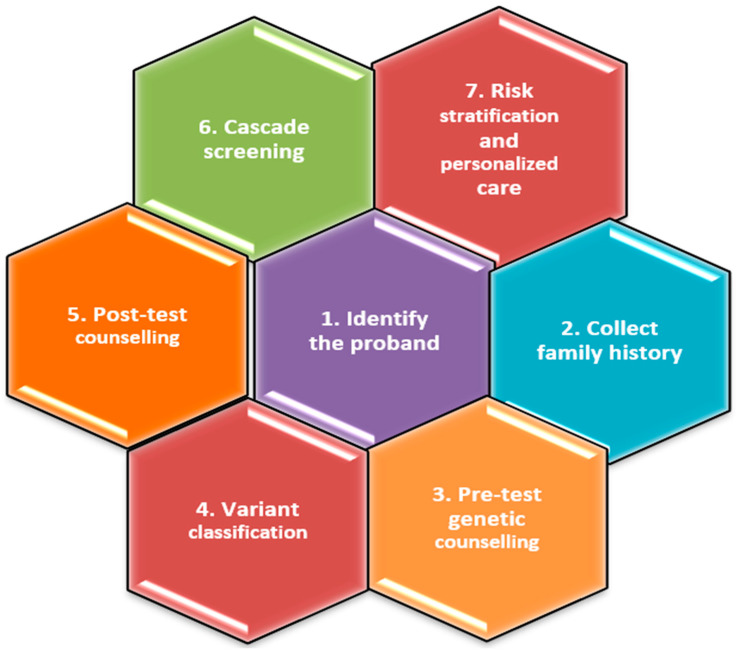
A practical approach for genetic testing in hereditary dilated cardiomyopathies.

Genetic testing is valuable for both the proband and their family members since it provides crucial information about the likelihood of DCM recurring or being transmitted [3,23,43]. A systematic methodology for the genetic assessment of FDCM is presented in Figure 7.

Family History. The initial stage in the genetic assessment of individuals suspected of having genetic DCM involves the identification of the proband and obtaining a comprehensive family history. Providers should construct a genealogical chart spanning at least three generations in order to determine the pattern of genetic inheritance, such as autosomal dominant/recessive or X-linked [40]. It is important to document the cause and details surrounding the death of any family member who passed away before the age of 50. An elaborate genealogical chart aids in assessing the first likelihood of genetic testing and identifying other relatives who may be susceptible to the condition. Prior to undergoing genetic testing, it is crucial to engage in genetic counselling.

Genetic counselling is assisting patients in comprehending the physical, psychological, and familial consequences of genetic abnormalities. Genetic counselling is often categorized into pre-genetic and post-genetic testing considerations [39].

Prior to conducting a genetic test, it is crucial for healthcare practitioners to engage in pretest counselling with patients. During this counselling session, physicians should thoroughly explain the procedure, advantages, and limits of genetic testing, as well as the expected results [33]. Other factors to consider before conducting the test include selecting the suitable genetic test and determining the most suitable individual to be tested.

Post-test counselling is crucial for ensuring precise result interpretation, which includes clarifying variants of uncertain significance (VUS) and any unexpected findings [40]. The effort to determine a relationship between gene variants of unknown significance and a phenotype of the disease is important not only for cardiovascular research but also for the clinical genetics of patients and their families. Combined variants in previously unreported genes related to cardiomyopathy might also play a significant role in affecting clinical, morphometrics, or myocardial mechanics parameters [45].

Additionally, after the test, it is crucial to start cascade screening of family members if necessary and offer psychosocial assistance to patients and families as required. Providers must possess a high level of skill in genetic interpretation, since it is a complex task. This is due to the fact that genetic tests do not have a simple “yes” or “no” result, and assessing pathogenicity is particularly problematic. This difficulty arises from the variability in how genes are expressed and the inadequate manifestation of hereditary traits [39]. A negative genetic test does not provide certainty that the condition is not hereditary, but rather indicates that no mutation was identified by the test [39]. A variant of uncertain significance (VUS) likewise has a broad range of confidence on its possibility of being pathogenic, ranging from 10% to 90% [34]. Hence, it is crucial for cardio-vascular genetic specialists to reassess outcomes, particularly those classified as VUS, in light of the patient’s clinical circumstances. This is especially important if the patient be-longs to a racial or ethnic group that is not well-represented in existing genomic data-bases, or if there is strong suspicion that a variant may cause a malfunction in the protein. Moreover, genetic testing frequently results in the discovery of incidental findings that are unrelated to the original reason for ordering the genetic test [46,47]. This phenomenon is more prevalent in genetic testing methods that are not specifically focused on a particular target. These methods include whole-genome or exome sequencing, as well as genetic panel testing. The challenges arise from the ethical considerations surrounding the dis-closure of incidental results, particularly when a significant number of these findings do not have practical implications for clinical management. The American College of Medical Genetics and Genomics issued clarifications in 2021 on whether incidental discoveries have actionable outcomes and hence should be addressed during genetic counselling sessions before and after genetic testing [46,47]. The mentioned genes associated with DCM include LMNA, filamin C (FLNC), PAG3, desmin, and RBM20 [48,49,50,51].

### 5.1. Benefits of Genetic Testing

Genetic testing is mostly valuable for identifying family members who are at risk and does not directly benefit the individual being tested (proband), but it can assist in deter-mining the cause of the disease [52]. Hence, the main advantage of genetic testing is the ability to conduct cascade screening of family members, which has the potential to detect subclinical illness [40,53]. Additional advantages encompass distinguishing illnesses that exhibit comparable phenotypic and morphological characteristics, making predictions about disease outcomes, classifying individuals based on their phenotypic risk, enabling early intervention, and providing personalized advice [39,53,54,55].

The use of genetic testing in DCM is anticipated to expand with increasing knowledge. 

### 5.2. Genetic Testing on the Proband

Conducting genetic tests on the reference patient who is the most probable person affected provides the greatest likelihood of identifying a pathogenic genetic mutation [3]. Choosing the appropriate array for a certain patient is crucial due to subtle variants.

Genetic panel testing is the predominant technique of testing [39]. Due to recent progress in sequencing technology, the process of including genes into these panels has be-come more convenient and cost-effective while maintaining the accuracy and precision of the results [19]. Genetic panel testing is highly beneficial in cases with DCM due to the presence of genetic diversity and the involvement of numerous potential genes [55]. Regular updates to genetic panels are crucial to ensure the inclusion of the latest medically actionable variants [46,47]. The introduction of next-generation sequencing has greatly reduced the expenses associated with whole-genome sequencing or whole-exome sequencing, making them viable options in clinical environments [5]. 

Whole-genome sequencing is considered the most reliable method for sequencing the entire genome. It provides comprehensive information about several forms of genetic variants, such as single nucleotide polymorphisms (SNPs), insertions–deletions (indels), splice-site variants, and genomic rearrangements [54]. Whole-genome sequencing is especially advantageous in the setting of dilated cardiomyopathy because it can identify dis-ease-causing genetic variants that may be located outside of the exon regions [55]. Thus, whole-genome sequencing can be valuable in cases when genetic panel testing fails to provide answers and there is a strong clinical suspicion of inherited DCM.

Genetic testing on patients with suspected hereditary DCM is usually performed using commercially available next-generation sequencing arrays that are efficient in detecting polygenic diseases. Inherited DCM is characterized by its diversity, with over 100 genes being involved [5]. The presence of genetic diversity in DCM has led to the development of a concept known as the “final common pathway.” This hypothesis suggests that many genetic variants impact diverse processes, which ultimately contribute to a shared final phenotype [56,57,58]. Typically, a genetic mutation alone is insufficient to trigger a DCM phenotype unless there is a second insult, such as hemodynamic, toxic, or stress factors. This concept is known as the “two-hit” theory [58]. It is crucial to acknowledge that several genetic variants linked to DCM exhibit substantial similarities with other cardiac disorders, such as arrhythmogenic ventricular cardiomyopathy (AVC) and arrhythmogenic right ventricular cardiomyopathy (ARVC). The presence of heterogeneity leads to a decrease in the success rate of genetic testing, which has been previously reported to be 16% in individuals with familial DCM [59]. Nevertheless, as the use of next-generation sequencing has grown, the effectiveness of genetic screening in identifying diagnoses has expanded, presently including a range of 16 to 46% [48,55,58,59,60,61].

### 5.3. Prenatal Genetic Testing

Identifying a disease-causing mutation in a potential parent can also be utilized to provide information for prenatal genetics, specifically pre-implantation genetic diagnosis. The process involves in vitro fertilization with the selection or modification of embryos [62]. This is a highly invasive procedure that involves many stages, including oocyte retrieval, in vitro fertilization, genetic testing of embryos, and implantation of embryos that do not have pathogenic variants. It is important for all patients who have a strong diagnosis with a clearly identified disease-causing or potentially disease-causing genetic mutation to be informed about this possibility. Chorionic villus sampling and amniocentesis are alternative methods used for prenatal genetic diagnosis. Given the multitude of choices available, it is imperative that patients receive comprehensive pre-test genetic counselling to guarantee they possess the necessary knowledge and assistance to make a well-informed choice. This encompasses the ability to comprehend the possible consequences of test results, examine the decision-making process in relation to the results, and facilitate communication with other family members who may be at risk [43].

### 5.4. Genetic Testing on the Relatives of the Proband

If a disease-causing genetic mutation is identified in the patient of interest, it is advisable to perform cascade genetic testing on the relatives [16]. 

Relatives who present the inherited DCM undergo investigations to stratify their risk and to establish the strategy of management.

Relatives who are negative for the illness but have a detected disease-causing or possibly disease-causing genetic mutation should have regular and frequent screenings. Un-affected carriers should receive counselling on the symptoms and signs of early-stage DCM. They should also undergo more frequent clinical surveillance, with check-ups scheduled every 1–3 years as recommended by the European Society of Cardiology guide-lines [5] or every 3–5 years according to the American Heart Association guidelines [16]. 

Relatives without an identified pathogenic variant still regarded susceptible to the disease, however, are recommended to take part in clinical screening. Typically, it is advised to undergo this procedure every two years; however, the frequency may be modified according on factors such as age and risk level [43]. The lack of an identifiable genetic mutation does not automatically indicate the absence of a genetic cause for DCM. The potential explanations are as follows: (a) a gene associated with DCM that is not included in the gene panel used for testing, which could be due to recently discovered disease genes or the absence of gene identification; (b) DCM may not have a purely monogenic cause; there may be a pattern of polygenic disease within families [44]. In addition, advancements in genetic knowledge might require families to undergo genetic re-evaluation in order to identify new pathogenic variants [63].

Family members who do not carry the disease-causing genetic mutation and are not regarded susceptible to the disease might be given reassurance and released from further screening [64].

## 6. Genes Strongly Associated with DCM and Potential Therapeutic Implications

The area of genetic testing has made significant progress in recent decades. During these years, several variants in more than 60 potential disease genes have been documented. However, this wide-ranging list has been narrowed down to 12 genes that have strong evidence linking them to the etiology of DCM, as determined by systematic analysis [3,4,5]. 

**Figure 8:**
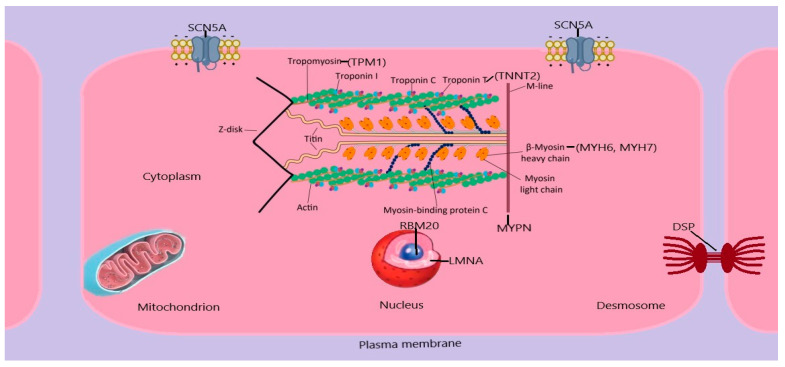
Cellular proteins linked to DCM. Abbreviations: DSP, desmoplakin; LMNA, lamin A/C; MYHN, myosin heavy chain; MYPN, myopalladin; RBM20, RNA binding protein 20; TNNT, cardiac muscle troponin T2; TPM1, α-tropomyosin 1; SCN5A, sodium channel protein type 5.

DCM exhibits genetic heterogeneity with a substantial number of cases manifesting as monogenic disorders, while a more complex genetic architecture is present in a number of cases. The majority of monogenic types of NSDCM have an autosomal dominant pat-tern of inheritance. The genes linked to GDCM are involved in several biological processes, such as sarcomere components, cytoskeletal and desmosomal proteins, and mitochondrial proteins, among others, as shown in Figure 8. The genetic etiology of other cardiomyopathies, such as hypertrophic cardiomyopathy, is less diverse compared to this. This indicates that NSDCM might be a common endpoint for multiple pathways [5].

Furthermore, there is a notable allelic heterogeneity, where several mutations within the same gene might result in a comparable phenotype. It is also important to mention that multiple variants of the same gene can induce different morphological characteristics [44].

Several genetic variants in DCM demonstrate partial and age-related penetrance, as well as heterogenous expressivity. Penetrance refers to the percentage of individuals with a certain genetic variant who exhibit the illness. Incomplete penetrance indicates that not all individuals with the genetic variant have the disease. Penetrance in GDCM is generally age-dependent. This means that an individual inherits the genetic mutation responsible for DCM from birth, but the symptoms of DCM usually do not appear until middle age (over 40 years old) or may not appear at all [3,7].

The level of penetrance for various genetic DCM variants is still unclear and is being actively researched. Heterogeneous expressivity means the presence of differences in both the severity and spectrum of clinical characteristics that are observed in individuals with a certain genetic condition. For instance, if we take into account two subjects from the same family who have the exact same genetic variation, one of them may experience severe DCM, which is indicative of heart transplantation, while the other one may only exhibit minimal symptoms. This suggests that there is a potential for other genetic, epigenetic, or external factors to contribute to the observed characteristics [39,63].

It is increasingly apparent that several genes can be linked to certain characteristics, such as arrhythmic forms of familial DCM, with a high risk of sudden cardiac death. Identifying these alarming phenotypes is crucial and should prompt immediate consideration of genetic testing [5].

Pathogen mutations can occur in genes that code for a miscellaneous proteins found in the cardiomyocyte sarcomere, cytoskeleton, and nucleus. Autosomal recessive, mitochondrial, and X-linked inheritance have also been documented [64,65].

Genes mutations strongly associated with non-syndromic DCM are described further.

### 6.1. TTN

The main genetic cause of familial DCM is represented by dividing mutations in the TTN gene which encodes the titin protein. These mutations are present in 15–20% of instances [6]. The mode of inheritance is autosomal dominant. Titin has a significant role in the structure and function of the sarcomeres of the adult myocardium [15,16]. Titin proteins have several functions, such as affording structural and regulatory support. They also have a crucial role in the contractility of skeletal muscles [66]. Truncation mutations in the TTN gene (TTNtv) are highly common in DCM, accounting for around 25% of familial and 18% of sporadic DCM cases [36,57,67]. TTNtv is distinguished by frequent occurrences of arrhythmias, particularly atrial fibrillation and ventricular arrhythmias [68,69].

Those diagnosed with dilated cardiomyopathy (DCM) and with a positive TTNtv (titin truncating variant) status are at a considerably increased risk of experiencing persistent ventricular tachycardia in comparison to those with a negative TTNtv status [70]. Research indicates that in a certain proportion of patients, TTNtv may have a synergistic interaction with other variables such as cardiotoxic chemotherapy or pregnancy. This suggests that addressing these aggravating factors might potentially result in significant improvement [71].

As our comprehension of gene mutations advances, the possibility of using genetic engineering for therapeutic purposes becomes evident.

Promising treatment options might arise from techniques such as reverse-mediated exon skipping, targeted therapy, and genome-editing technologies [72,73,74].

### 6.2. LMNA

The second most prevalent reason for hereditary DCM and possibly the most severe one consists in alterations in the LMNA gene, which encrypts proteins Lamins A and C of the nuclear envelope [3]. The prevalence of Lamin A/C variants in individuals diagnosed with DCM is around 6%, and the mode of inheritance is autosomal dominant [6,75,76].

Individuals with pathogenic LMNA mutations have a significantly increased risk of experiencing sudden cardiac death as a result of malignant arrhythmias. Additionally, their overall prognosis is unfavorable [49,77]. Laminopathy frequently involves frameshift mutations that are commonly linked to heart disease. Splice site mutations, on the other hand, are an autonomous risk factor for sudden cardiac death. Non-missense mutations, such as deletions, mutations affecting splicing or truncations are significant risk factors for malignant ventricular arrhythmias. A precise mechanism by which DCM is induced by LMNA mutation is still not fully understood. However, three theories, including mechanical, gene expression, and cytotoxicity, have been suggested to elucidate the myocardial dysfunction related with it [5,6,49,77].

The gene expression theory suggests that faulty Lamins hinder the process of signal transduction and the organisation of chromatin, thereby affecting signal transduction, which is a crucial factor in the development of LMNA-associated dilated cardiomyopathy. This immediately impacts and interrupts the process of gene transcription and other in-ternal signalling pathways, resulting in a notable increase in myocardial fibrosis. This, in turn, causes LV dysfunction and ultimately heart insufficiency [78,79].

Because there is a connection between heart block, bradycardia, and the risk of sudden cardiac mortality in LMNA-related DCM, it is suggested to use Implantable Cardioverter Defibrillators (ICDs) [80].

Additional pathways associated with the LMNA gene have been investigated as potential therapeutic targets. These include the use of rapamycin/rapalog to block mTOR and MEK1/2 kinase pathways, inhibiting the activation of brominated domain protein 4 (BRD4), and targeting the destruction of LINC complex protein SUN1 to counteract LMNA mutations [81,82,83,84,85].

Currently, there is no particular and efficacious therapy available.

### 6.3. MYH7

The MYH7 protein is essential for supplying energy to cardiomyocytes and regulating the levels of Ca2+ both within and outside these cells [85,86]. MYH7 mutations are responsible for 1% to 5.3% of inherited DCM [87]. The mutations mostly consist of missense variants, which are transmitted in a dominant pattern on the chromosomes. These mutations have a high penetrance in families [86,88]. Genetic mutations in the MYH7 gene can impair the structural and functional integrity of the sarcomere, leading to abnormalities in the contraction of the myocardium [86]. Atrial fibrillation and atrial fibrosis are recognized as first clinical signs of MYH7-related cardiomyopathy, offering significant knowledge for disease identification [87,88,89]. It has been documented that the combination of mutations in MYH7 and TNNT2, MYH7 and LAMA4, or MYH7 and TPM1 can lead to the development of severe DCM [90,91,92].

These findings emphasize the need of conducting thorough screening of genes associated with dilated cardiomyopathy (DCM), even after discovering a single mutation that causes the condition. Research has demonstrated that the length of telomeres in mice can provide defence against heart disease in people. Genetic alterations in proteins that play a crucial role in the functioning of cardiomyocytes, such as MYH7, TTN, and MYBPC3, result in the reduction in telomere length. Therefore, substantial reduction in telomere length can be used as a biomarker to indicate accelerated ageing of cardiomyocytes in hereditary hypertrophic cardiomyopathy and DCM [93,94].

TRF2, also known as Telomere Repeat Binding Factor 2, has been shown to inhibit the erosion of telomeres, leading to the enhancement of cellular morphology, suppression of DNA damage response, and prevention of premature cell death [95].

### 6.4. BAG3

Bcl2-associated athanogene 3 (BAG3) encodes for a protein that prevents cell death and is found on the Z disc of myotomes. BAG3, a member of the anti-apoptotic BAG protein family, is highly expressed in the heart [96,97].

BAG3 is crucial in preventing cell death, maintaining the balance of proteins, regulating the integrity of mitochondria, controlling cardiac contraction, and reducing the occurrence of arrhythmias [98,99]. These mutations of BAG3 are present in about 3% of hereditary DCM and the mode of inheritance is autosomal dominant [6].

Research suggests that every identified or likely harmful genetic variants affect either the WW domain, the Ile-Pro-Val (IPV) domain, or the BAG domain. The three protein domains have important functions in BAG3 action in the heart [99,100].

Research indicates that DCM caused by BAG3 mutations is often observed at an early age in the majority of patients. There is a significant likelihood of the condition advancing to end-stage heart failure, and men generally have a poorer prognosis [101].

BAG3, functioning as a cochaperone protein, forms interactions with high-molecular-weight heat shock proteins that are ATP-dependent, as well as small heat shock proteins (sHSPs) that are ATP-independent. These interactions occur within huge multichaperone protein complexes that have diverse functions. Once BAG3 is eliminated, the levels of impacted sHSPs decrease as a result of protein instability [102,103].

The recruitment of macrophages controlled by BAG3 can help maintain protein homeostasis. However, in hearts lacking BAG3, the process of autophagic flux is reduced, potentially leading to the formation of misfolded protein aggregates [104]. As a result, there is a rise in the amount of proteins that cannot dissolve in a liquid, which speeds up the ageing of cells. MicroRNAs, also known as miRNAs, are short non-coding RNAs that play a role in regulating the development and functionality of the cardiovascular system through epigenetic mechanisms. They are typically composed of 20–25 nucleotides.

The misregulation of their expression is closely linked to the pathogenesis of several cardiovascular disorders [104,105].

Research shows that the simultaneous presence of miR-154-5p and miR-182-5p has diagnostic significance in dilated cardiomyopathy (DCM) in individuals with BAG3 gene mutations [106]. Genetic compensation, which refers to the transcriptional adaptation of gene expression in response to deleterious gene mutations, has been found in zebrafish lacking the BAG3 gene. This compensatory mechanism serves to safeguard the heart and skeletal muscles from injury. This biological phenomenon may also be present in certain individuals with BAG3 mutations [107]. Additional exploration of the pertinent molecular pathways may provide novel perspectives for the advancement of therapeutic approaches.

### 6.5. RBM20

The main role of the RNA-binding motif protein 20 (RBM20) is to regulate splicing. It is mainly found in the heart and skeletal muscle, where it controls both constitutive splicing and alternative splicing of pre-messenger RNA. RBM20 gene mutations, primarily characterized by missense mutations that modify conserved residues, are a major contributor to DCM [108,109].

These mutations are responsible for around 3% of all occurrences of dilated cardio-myopathy and transmitted in an autosomal dominant mode [110].

The pathophysiology of these RBM20 mutations arises from a mix of functional loss and harmful functional increase [111]. The TTN gene is the most important among those controlled by RBM20 [112,113]. Reduced RBM20 activity also leads to changes in the expression of protein subtypes that support muscle shape and cardiac function, including CAMKIIδ, LDB3, and CACNA1C. These modifications have the potential to cause variations in the biomechanics, electrical activity, and signal transduction processes, which can ultimately result in cardiomyopathy, fibrosis, arrhythmia, and sudden death [113,114].

Individuals diagnosed with dilated cardiomyopathy (DCM) who possess RBM20 mutations frequently have compromised cardiac function and are prone to experiencing atrial fibrillation, ventricular arrhythmia, and sudden cardiac death [115]. All-trans retinoic acid (ATRA) has been discovered as a possible regulator of RBM20. Research has demonstrated that ATRA has the ability to enhance RBM20 expression and partially re-verse the in vitro dilated cardiomyopathy (DCM) characteristics. Hence, the use of drugs to increase RBM20 expression might be a potentially effective treatment approach for individuals with dilated cardiomyopathy who have RBM20 mutations on one of their gene copies (heterozygous). The majority of RBM20 mutations are concentrated in a region that is rich in arginine/serine, indicating that targeted gene editing techniques such as adenine base editing and primer editing might potentially be used as therapies [115].

### 6.6. FLNC

The filamin family consists of three isomers: filamin A (FLNA), filamin B (FLNB), and filamin C (FLNC) [116]. FLNC is predominantly found in skeletal and cardiac muscle [117]. FLNC is essential for controlling cellular mechanics, the organization and orientation of Z-disks, and the connections between myofibrils in mammalian hearts [117,118,119,120,121,122,123]. Cardiomyocytes lacking FLNC can result in fetal mortality. In addition, adult mice lacking FLNC have fast and severe DCM within a period of two weeks [120]. In adults, mutations in FLNC are found in about 2–4% of DMC and have an autosomal dominant mode of inheritance [6].

These findings emphasize the important function of FLNC in both the development and functioning of heart muscle cells in both young and adult individuals. A truncation mutation in the FLNC gene (FLNCtv) is strongly linked to DCM [121]. Individuals diagnosed with FLNCtv commonly have enlargement of the left ventricle along with impaired contraction of the heart during systole and the presence of fibrous tissue in the myocardium. Ventricular arrhythmias are common, and families with these mutations have a high occurrence of sudden cardiac death [122,123,124]. β-catenin (CTNNB1) has been identified as the downstream target of FLNC by use of co-immunoprecipitation and proteomic studies.

FLNC does not have the ability to cause the movement of CTNNB1 into the nucleus. However, this lack of nuclear translocation of CTNNB1 leads to the activation of the platelet-derived growth factor receptor-α (PDGFRA) pathway. Blocking PDGFRA can partially revert the abnormal gene expression pattern of FLNC in patient-specific cardiomyocytes, which is associated with cardiac insufficiency and arrhythmia [124]. Hence, blocking this route might be a promising treatment strategy for FLNC-related cardiomyopathy.

### 6.7. SCN5A

The sodium channel (SCN) 5A gene, located on human chromosome 3p22, is responsible for producing the α subunit Nav1.5, which forms the pore of the cardiac sodium channel. The SCN5A/Nav1.5 gene is mostly active in the atrial and ventricular muscles of the heart, as well as in the His bundle, bundle branches, and Purkinje fibres [125]. Ap-proximately 2% of individuals with DCM have SCN5A gene mutations, and these have an autosomal dominant mode of inheritance. They can lead to a functional impairment of the sodium channels in the heart [126]. The precise biochemical mechanism by which these mutations lead to ventricular dilatation and dysfunction has not been completely comprehended [127]. SCN5A mutations can disrupt the connection between Nav1.5 and other components of the complex, resulting in structural abnormalities and impairment to contractile function [128].

The voltage-gated ion channel has two mutations (R222Q and R225W) in the voltage sensor domain. These mutations are believed to produce gated hole currents that might potentially be associated with arrhythmia and ventricular dilatation in humans [128]. Occult myocardial damage can occur due to the dysfunctional activity of the mutant SCN5A immune sensor [129]. DCM often displays age-related penetrance, where the characteristics become more noticeable as individuals become older [130]. From a clinical perspective, it frequently presents as severe irregular heart rhythms, such as atrial fibrillation and ventricular tachycardia, as well as conduction block.

Commencing the use of sodium channel blockers can effectively reduce the occurrence of severe illness and death [131].

Studies have also shown that the mRNA stabilizing protein HuR safeguards SCN3A by attaching to the 5′-UTR mRNA, therefore preventing its degradation. To lessen the likelihood of arrhythmia, one can enhance the stability of mRNA in order to maintain de-creased expression of SCN5A [132].

### 6.8. TNNC1, TNNT2

The TNNC1 gene is responsible for encoding cardiac troponin C (cTnC) in heart tis-sue, while the TNNC2 gene is responsible for encoding cardiac troponin T (cTnT) [133]. The trimer filament Tn complex, which plays a role in muscle contraction, is created by the fusion of cTnT, cTnI, and cTnC [134]. Point mutations in TNNC1 can impact the function of cTnC in two manners: by altering its ability to connect with Ca2+ or by influencing the interaction between cTnC and its binding partner [135]. Approximately 6% of familial DCM cases [136] have troponin complex mutations. The frequency of mutations in the TNNC1 gene is around 1% [137]. The prevalence of TNNT2 mutations in dilated cardio-myopathy (DCM) is around 3% [138]. The mode of inheritance is autosomal dominant. Individuals carrying TNNC1 gene mutations are usually identified at a younger age and have an increased likelihood of encountering life-threatening incidents. These incidents commonly present as severe systolic heart failure at an early stage, requiring the need for heart transplant surgery [127,139]. Studies suggest that a diminished responsiveness of myofilaments to Ca2+ is a crucial factor in the development of filament-associated DCM. Increasing the responsiveness of myofilaments to calcium ions in the first phases of DCM might potentially be a successful approach for therapy [140]. Xin actin-binding repeat containing proteins (XIRPs) are a set of proteins that are particular to rhabdoms. The XIN protein, which is produced by the XIRP1 gene and is sometimes referred to as HXin-α or CMYA1, is a gene that is particular to the rhabdom and belongs to the XIRP family. In-creased expression of the repetitive isomer XINB can improve the remodelling of DCM caused by TNNT2-ΔK210 mutations in mice. This can partially reverse the enlargement of the heart, impaired contraction of the heart, and the formation of fibrous tissue in the heart. Thus, XIN has the potential to be a therapeutic target [141].

### 6.9. DES

The major intermediate filament (IF) protein, desmin, is encoded by the DES gene in human heart and skeletal muscle [142]. Desmin functions as a fundamental element of the extramuscular cytoskeleton, creating a complex framework that surrounds the Z disc of the myofibril. This structure serves as a connection between neighbouring myofibrils and the myofibril apparatus to the nucleus, submuscular cytoskeleton, and cytoplasmic organelles including mitochondria [143]. Desmin is the IF protein that is most commonly found in muscle-specific tissues and cardiomyocytes, with a high level of expression [144]. The occurrence of desmin gene mutations in individuals with DCM is below 1.6%. The mode of inheritance is autosomal dominant [145]. The majority of DES variants are missense mutations located in the central domain [146]. Around 74% of individuals with DES mutations experience heart symptoms, and around 50% develop cardiomyopathy, with dilated cardiomyopathy (DCM) being the predominant form [145]. Studies using mice with a deletion of the DES gene have shown that desmin deficiency only affects the anatomy of the heart, but also leads to significant abnormalities in the way the heart processes glucose, fatty acids, and amino acids in its metabolism [146,147,148,149]. Hence, it would be wise to avoid medicines that might potentially worsen mitochondrial function in individuals with desmin deficiency [150]. The α-crystallin B-chain, also known as αB-crystallin, is produced by the Desmin and CRYAB genes. It has the ability to potentially interact in a compensatory manner to provide protection for the heart. Increased production of heart-specific αB-crystallin enhances mitochondrial dysfunction in mice models lacking desmin, indicating a promising novel therapeutic strategy [151].

### 6.10. PLN

Phospholamban (PLN) is a protein located on the membrane of the sarcoplasmic and endoplasmic reticulum (SR/ER) and is in charge for the encoding of a key regulatory protein linked to the cycling of Ca2+. It acts as the main mediator of beta-adrenergic actions, resulting in an increase in cardiac output [152].

Out of all the PLN mutations that have been identified, the PLN-R14DEL mutation is the most common [153,154]. The incidence rate of mutations in the PLN gene as a cause of cardiomyopathy is less than 1%, the inheritance mode is autosomal dominant [155,156].

A comprehensive multicenter investigation with a prolonged period of observation was conducted on individuals carrying the PLN mutation. The study revealed that PLN-R14DEL mutant carriers frequently experienced early ventricular arrhythmia and advanced heart failure, leading to a substantial rise in mortality rates within the cohort [157]. From the viewpoint of a cardiomyocyte, it is acknowledged that PLN R14del has a major impact on its function. Studies have demonstrated that the R14del mutation leads to the accumulation of PLN protein, increase in the unfolded protein response, disruption of calcium control, and impaired contractile and metabolic performance [158]. Although the genetic cause of PLN R14delcardiomyopathy is clearly recognized, the specific molecular process that leads to its development is yet unknown [159]. Low-voltage electrocardiograms are more prevalent in women with PLN mutations, although their prognostic significance is greater in males [160]. Eplerenone medication has been suggested as a potential method to hinder or delay the advancement of the illness in individuals who carry presymptomatic PLN mutations.

Additional multicenter randomized double-blind studies are underway to validate this finding [161]. As our comprehension of the PLN-R14del mutation process advances, precision medicine, which includes gene editing and targeted gene therapy, may emerge as a novel approach for future treatment [159,162,163].

Nevertheless, the majority of ongoing research is still in the animal experimentation stage, and it is uncertain whether these discoveries will be relevant to human subjects [164].

### 6.11. DSP

The desmoplakin (DSP) gene was determined to be a significant contributor to DCM, not a definite cause (4). Research indicates that DSP mutations are specifically seen in adults with DCM, but the % of DCM caused by pathogenic variants in the gene is unknown. The mode of inheritance is autosomal dominant [165].

Palmoplantar keratoderma can indicate the presence of dilated cardiomyopathy (DCM) linked with a desmoplakin (DSP) mutation at an early stage [166]. Individuals who carry DSP mutations have a greater likelihood of experiencing arrhythmia episodes, comparable to those with LAMA variants, even if they do not have noticeable left ventricular dysfunction or dilatation [167]. Desmoprotein cardiomyopathy has been categorized as an arrhythmic cardiomyopathy caused by mutations in the DSP gene, according to studies. This syndrome is defined by sudden episodes of damage to the heart muscle, the development of fibrosis in the left ventricle before a decrease in its ability to contract, and a high occurrence of abnormal heart rhythms [168,169]. Common electrocardiogram (ECG) abnormalities consist of limb lead QRS depression (peak amplitude less than 0.5 mV) and T-wave inversion in the lateral or inferior leads [168,170]. In arrhythmogenic cardiomyopathy (ACM) resulting from DSP variation, cardiomyocytes secrete a significant quantity of inflammatory cytokines and chemotactic molecules [171].

Examination of left ventricular myocaroma in individuals with DSP has also shown the presence of inflammatory infiltration and scarring [172]. Therefore, inflammation is seen as a crucial characteristic of the condition, and controlling the pathways that trigger inflammation might potentially serve as a novel treatment approach for desmosome-mediated cardiomyopathy.

### 6.12. Mitofusin (MFN)

Franco et al. conducted a genetic screening of individuals with cardiomyopathy and discovered an uncommon mutation in the mitofusin 2 gene, known as R400Q, which was shown to be more prevalent in this particular population. Mutations in the gene encoding mitofusin 2 have been predominantly associated with nerve damage, but recent studies have demonstrated that interfering with the scheduled degradation of mitochondria can result in the development of dilated cardiomyopathy. When injured, mitochondria are repaired by a process that involves fusing with and exchanging contents with other mitochondria, this process being facilitated by mitofusin proteins. These are valuable findings on the link between mitofusin 2 function and Parkin-mediated mitophagy, showing that a combination of mitophagy and mitochondrial fusion defects can lead to genetic dilated cardiomyopathy [173].

## 7. Genetic Risk Factors and Prognosis

There is significant interest in using genetic markers to assess prognosis and aid in risk stratification for individuals with DCM, in addition to predicting their likelihood of developing the condition [6]. Genetic testing holds significant potential to enhance precision medicine by providing valuable information for risk assessment and tailored treatment approaches.

Genotype-based classification is becoming more supported as a method for stratifying the risk of individuals with cardiomyopathies as opposed to using phenotype-based classification [174].

For those who have a positive genotype but do not display any physical signs of the condition, there is presently no strong evidence to support a specific approach for man-aging their condition. Furthermore, there are no treatments available that have been proven to prevent the development of DCM in these individuals [57]. It is crucial to provide genetic counselling and effectively control cardiovascular risk factors in this particular group.

Individuals who possess high-risk genetic variants such as LMNA, FLNC, desmoplakin (DSP), and plakophilin-2 are at risk for malignant ventricular arrhythmias and sudden cardiac death, and should be evaluated for implantable cardioverter defibrillator (ICD) placement, regardless of their physical characteristics or left ventricular dilatation and systolic performance [175,176]. At present, the Heart Failure Society of America recommends considering the use of an implantable cardioverter defibrillator (ICD) for patients who have a left ventricular ejection fraction (LVEF) greater than 35% and also have an LMNA mutation [16]. The Heart Rhythm Society includes the possibility of using an implantable cardioverter defibrillator (ICD) in individuals with a left ventricular ejection fraction (LVEF) of less than 45% [121]. In the 2022 European Society of Cardiology guidelines for prevention of sudden cardiac death, it is recommended that patients with dilated cardiomyopathy (DCM) and a left ventricular ejection fraction (LVEF) of less than 50% receive primary prevention implantable cardioverter defibrillators (ICDs) if they have two or more of the following risk factors: syncope, late gadolinium enhancement on cardiac magnetic resonance imaging (MRI), inducible sustained monomorphic ventricular tachycardia during programmed electrical stimulation, and pathogenic variants in the LMNA, PLN, FLNC, and RBM20 genes [49].

Familial DCM can be a progressive condition, with a substantial number of individuals advancing to end-stage heart failure and needing cardiac transplantation [1,5,20,40,171,172,173,174,175,176,177,178,179,180,181,182,183,184,185]. Single nucleotide polymorphisms (SNPs) can provide valuable information for the categorization of this risk. In a large-scale genome-wide association study (GWAS), researchers found that a single nucleotide polymorphism (SNP) located on chromosomal band 5q22 was associated with a 36% higher risk of mortality in individuals diagnosed with heart failure [177].

Nevertheless, further data are needed to support the regular use of genetic testing for predicting outcomes in hereditary DCM.

## 8. Management

### 8.1. Non-Specific Therapy

There are no specific clinical studies focused on pharmacological treatment for heart failure in inherited DCM, but all the significant trials on heart failure with decreased ejection fraction did involve patients with non-ischemic cardiomyopathy, and it is likely that some of these patients had genetic causes for their condition.

Familial DCM is an important cause of severe heart failure [1,4,5]. Although the reported death rate at 5 years is of 50%, the general prognosis is improving. This can be attributed to improved management of cardiovascular risk factors, earlier detection of the condition, and advancements in heart failure treatment, including heart transplantation [180,181,184].

The current approach to treating familial DCM involves using guideline-directed medicinal interventions that try to prevent or decelerate the progress of heart failure [4,5]. The drugs consist of the usual combination of beta blockers, angiotensin receptor–neprlysin inhibitors, sodium–glucose contransporter-2 inhibitors, and mineralocorticoid receptor antagonists [40]. Administering this medication to individuals with DCM caused by truncating TTN variants frequently lead to the reversal of pathological left ventricular remodeling [185].

Research has shed light on the effectiveness of neurohormonal blocking in heart failure patients categorized by genetic variants, which has contributed to the implementation of pharmacogenetics. The variation in individual responsiveness to β-blockade is likely due to genetic variants in the gene encoding the β-adrenergic receptor within the population. Polymorphisms in ADRB1 and GRK5, which encode the downstream G-protein-coupled receptor kinase 5, have an impact on the response to β-blockade [183]. Additional results indicate that amino acid changes in the β-adrenergic receptor, such as Arg389Gly, might potentially impact the response to β-blockade. This effect can be explained by the reduction of the renin–angiotensin–aldosterone pathway. Nevertheless, this polymorphism has not been linked to any change in morbidity or death in the sub-studies of the WOSCOPS trial [185] or the MERIT-HF trial [186].

Genes that encode angiotensin II and its accompanying receptors have a role in the development of heart failure and are targeted by antagonistic medication according to medical guidelines. These genes have polymorphisms that result in different responses to treatment. Evidence indicates that individuals with a homozygous DD genotype for the ACE gene, which increases angiotensin II levels, along with a polymorphism in AGTR1 (which encodes the type 1 angiotensin II receptor), may experience higher activation of the renin–angiotensin–aldosterone system and a poorer prognosis, even when treated with ACE inhibitors [187]. Research was conducted to observe individuals with congestive heart failure. It was shown that having at least one D allele of the ACE gene was linked to decreased survival rates, particularly for patients who were not receiving β-blocker medication. However, in another experiment where patients were categorized based on the existence of previously recognized pathogenic changes in AGTR1, no disparities in long-term mortality were found [188].

While the impact magnitude may not be significant, certain single nucleotide poly-morphisms (SNPs) have been discovered that elevate the risk of angio-oedema produced by ACE inhibitors [183,188]. In the clinical arena, routine testing for SNPs that are linked to variable responses to neuro-hormonal blocking is not commonly performed since there is a lack of clinical validity and usefulness.

The interplay between environmental variables and an individual’s genetic composition is of utmost significance. Environmental variables and triggers have the potential to influence the observable characteristics of DCM in a patient who already has a genetic susceptibility to the condition. Peripartum cardiomyopathy has been linked to both genetic susceptibility and the interaction between an underlying pathogenic variation and the physiological stress of pregnancy [14,189]. Furthermore, the cardiotoxicity of anthracyclines and other antineoplastic drugs may be influenced by genetic predisposition [190]. Upon thorough evaluation, it is evident that environmental triggers may be linked to a poorer prognosis if there is an existing genetic susceptibility to the illness [191]. Crucially, it is vital for both DCM patients and their families to focus on addressing changeable variables that contribute to cardiovascular disease and overall health. The conventional approach to managing hypertension, quitting smoking, preventing diabetes, addressing obesity, and moderating alcohol intake continues to be essential, since additional pathogenic factors affecting the heart are linked to poor outcomes [187]. A primary care setting handles the management of many of these concerns.

### 8.2. Specific Therapy

In general, pharmacological therapies in inherited DCM are not disease-modifying, with some exceptions.

A notable exception to this is the p.R222Q SCN5A mutation, which allows for the possibility of tailored treatment based on particular mechanisms [192]. This genetic mutation leads to the development of multifocal ectopic Purkinje-related premature contractions (MEPPC), atrial arrhythmias, and DCM as a result of increased activity in the heart sodium channels. The symptoms mentioned can be efficiently managed by using a sodium channel antagonist, such as flecainide. This type of treatment is considered a gene-specific therapy and is a significant example of precision medicine in familial DCM.

Additional gene-specific medicines that are currently being studied but have not yet been used in clinical practice include myosin activator medications (such as omecamtiv mecarbil) and the suppression of the mitogen-activated protein kinase signaling pathway in laminopathies [5].

Implantable cardiac defibrillator (ICD) treatment can be utilized as either primary or secondary prevention after experiencing life-threatening arrhythmia. Current guidelines employ genetic variants such as LMNA, FLNC, and PLN to categorize individuals based on their risk for malignant arrhythmias. These guidelines suggest the use of primary prevention ICDs for patients with a higher risk, even if their left ventricular ejection fraction (LVEF) is over the usual criterion of 35% [183].

Determining a genetic anomaly is also crucial in making judgements on the appropriate timing for heart transplantation. Patients with pathogenic or possibly pathogenic LMNA mutations frequently experience a swiftly deteriorating clinical condition and re-quire early consideration of cardiac transplantation.

Certain pathogenic variants have stricter guidelines. For instance, individuals with LMNA-associated DCM should avoid engaging in intense physical activity since it is linked to a higher risk of deterioration in heart function and death [193]. It is extremely advantageous and important to pay attention to the psychological well-being and receive genetic counselling from specialists who have been trained in this field. This should not be disregarded or underestimated.

The research of Franco [173] regarding the mutations of MSN2 that affect mitochondrial repair and replacement processes highlight future directions for studies into modifiers of genetic cardiomyopathies.

Contraception or pregnancy planning is crucial for pre-menopausal women with DCM. Women who test positive for a certain genotype and become pregnant should have thorough echocardiographic surveillance both during and after pregnancy to identify any indications of worsening after childbirth. Peripartum cardiomyopathy has the potential to reoccur earlier and with greater severity in subsequent pregnancies. Indeed, for certain women with confirmed DCM, pregnancy may be advised against due to its exceedingly precarious nature, which poses significant risks to both the mother and the fetus [194]. When it comes to contraception for individuals with cardiomyopathy, it is recommended to use barrier techniques or long-acting contraceptive methods that contain levonorgestrel.

For those who have a positive genotype but do not display any physical signs of the condition, there is presently no strong evidence to support a specific approach for man-aging their condition. Furthermore, there are no treatments available that have been proven to prevent the development of DCM in these individuals [195,196]. It is crucial to pro-vide genetic counselling and effectively control cardiovascular risk factors in this particular group.

## 9. Unresolved Issues and Future Directions

Over the past decade, there has been significant advancement in understanding and improving our knowledge of the genetic foundation of DCM. The objective for the next decade should be to utilize this information in order to develop specific treatments. There are some unresolved inquiries in our comprehension of the genetics of DCM. An enigma is in the very modest genetic diagnostic success rate, especially in cases of familial DCM. This presumably indicates situations with complex genetic structures that are more challenging to investigate—highly influenced by genetics, but not caused by a single gene. These theories encompass oligogenic and polygenic combinations, in which genetic susceptibility is influenced by the cumulative impact of numerous common genetic variants. This is in contrast to a dominant inheritance pattern driven by a single strong genetic component, as seen in Mendelian inheritance. These extra variants can have either additive effects, where the combined burden of these more frequent variants contributes to the disease, or non-additive interactions, where the more common variants affect the impact of a rare variant. Gene–environment interactions are expected to alter the impact of both uncommon and common variants [195,196].

## 10. Conclusions

Hereditary DCM remains a highly complex condition with broad phenotypic heterogeneity that includes both arrhythmogenic and non-arrhythmogenic forms. Determining a genetic anomaly is also crucial in making judgements on the appropriate timing for heart transplantation in individuals with heart failure.

The heart failure community must advance in the identification and validation of genetic markers that indicate the risk of DCM, the progression of the disease, and the response to therapy. This progress will ultimately lead to the realization of precision medicine in heart failure, with the potential for improved outcomes for patients and their families.
